# Water-in-Oil-in-Water Nanoemulsions Containing Temulawak (*Curcuma xanthorriza* Roxb) and Red Dragon Fruit (*Hylocereus polyrhizus*) Extracts

**DOI:** 10.3390/molecules26010196

**Published:** 2021-01-02

**Authors:** Niken Harimurti, Mohammad Nasikin, Kamarza Mulia

**Affiliations:** 1 Chemical Engineering Department, Faculty of Engineering, University of Indonesia, Depok 16424, Indonesia; niken.harimurti@ui.ac.id (N.H.); mnasikin@che.ui.ac.id (M.N.); 2 Indonesian Center for Agricultural Post Harvest Research and Development, Indonesian Agency for Agricultural Research and Development, Jakarta 12540, Indonesia

**Keywords:** betacyanin, curcumin, red dragon fruit, temulawak, w/o/w nanoemulsion

## Abstract

Hydrophobic curcumin in temulawak extract and hydrophilic betacyanin in red dragon fruit extract are high-value bioactive compounds with extensive applications in functional food. In this study, these extracts were encapsulated in water-in-oil-in-water (w/o/w) nanoemulsions as a delivery system using a two-step high-energy emulsification method. PGPR and Span 20 were used as lipophilic emulsifiers for the primary w/o emulsion. The most stable w/o/w formulation with the least oil phase separation of 5% *v*/*v* consisted of w/o emulsion (15% *w*/*w*) and Tween 80 (1.5% *w*/*w*) as hydrophilic emulsifier. The formulation was characterized by a 189-nm mean droplet diameter, 0.16 polydispersity index, and –32 mV zeta potential. The freeze–thaw stability may be attributed to the combination of low w/o emulsion content and high Tween 80 concentration in the outer water phase of the w/o/w nanoemulsions used in this study. The IC_50_ values of the nanoemulsion and the red dragon fruit extract were similar. It means that the higher concentration of curcumin in the nanoemulsions and the lower IC_50_ value of temulawak extract ensured sufficient antioxidant activities of the w/o/w nanoemulsions.

## 1. Introduction

The heightened awareness of the public about the risks involved in consuming unhealthy food and its association with lifestyle diseases, such as obesity, cardiovascular diseases, diabetes, and several types of cancer, has given rise to an increase in the production and consumption of nutrient-rich foods. Diseases are prevented by adopting a healthy lifestyle, including living in a pollution-free environment [1]. A valuable, naturally occurring bioactive phenolic compound that is present in food and widely used in the production of nutraceuticals and cosmetics is curcumin.

Curcumin is a hydrophobic, low-molecular weight polyphenol extracted from turmeric [2]. It is one of the primary curcuminoids that contributes to the characteristic yellow color of the extract obtained from temulawak (*Curcuma xanthorriza* Roxb), turmeric (*Curcuma longa* Linn), and white turmeric (*Curcuma zedoaria*). It has anti-inflammatory, antioxidant, antifungal, antiviral, antitumor, anti-cancer, anti-carcinogenic, and antimicrobial pharmacological attributes and therapeutic potentials [3,4]. Notwithstanding its health benefits, the application of this natural polyphenol in food and pharmaceuticals is limited by its exceptionally low bioavailability due to poor water solubility and instability in both in vivo and in vitro environments [4,5].

Betacyanins are natural red-violet, water-soluble nitrogenous pigments. Along with yellow betaxanthins, they comprise the constituents of betalains. Seven betacyanins have been found in red dragon fruit (*Hylocereus polyrhizus*): betanin, isobetanin, lhyllocactin, isophyllocactin, betanidin, isobetanidin, and bougainvillein-R-I [6]. They have also been proven to possess various antioxidant, anti-cancer, anti-lipidemic, and antimicrobial pharmacological attributes, among others [7]. Like curcumin, betacyanins have a short shelf life and low bioavailability [8]. Moreover, they tend to be unstable under certain external factors, namely, oxygen, temperature, light, pH, water activity, and enzymes [9,10]. 

Several studies have been carried out to overcome the limitations of curcumin and betacyanin. The poor cellular uptake of free curcumin and the enhanced stability realized by encapsulating it in nanoliposomes have also been reported [11]. The development of curcumin nanoemulsions has exhibited potential applications in the beverage industry. The encapsulation of curcumin and catechin in water-in-oil-in-water (w/o/w) double emulsion showed improved stability in simulated gastrointestinal fluid [5]. The microencapsulation of betalains with potato succinylated starch enhanced the stability of yogurt stored at 4 °C with a pH of 4.6 for 32 days [12]. The easiest way to prepare nanoliposome phospholipids in a research laboratory is through the thin-film hydration method. It improved the stability of betanin and showed higher DPPH (2,2-diphenyl-1-picrylhydrazyl) radical-scavenging activity than in gummy candies containing free betanin [8]. The betalain pigments encapsulated in w/o/w double emulsions showed high encapsulation efficiency (89.1%) and emulsion stability [10]. However, given that curcumin and betacyanin have similar antioxidative properties, the development of an edible delivery system for nutraceuticals tends to be quite challenging because of the differences in the solubility and potentials of novel food applications. The challenge that needs to be resolved is that all of the materials must be food-grade and have sufficient physical and chemical stability in conditions frequently encountered in the food industry [13,14]. Notably, the use of double emulsion systems for the delivery of low-fat diets, nutrients, and flavors has been cited as a feasible solution.

Double or multiple emulsions are defined as liquid dispersion systems in which the droplets of the first phase are dispersed into the second phase. Then, the droplets of the first emulsion are further dispersed into a third phase [13,14,15,16,17,18]. The two major types of double emulsions are w/o/w and oil-in-water-in-oil (o/w/o) emulsions. The w/o/w emulsions are preferably used in food applications because most edible materials constitute a continuous aqueous phase [10,13,14]. This emulsion system permits the simultaneous delivery of hydrophilic and hydrophobic compounds using a single carrier, enhancing the efficiency of treatment, and thereby resulting in situ synergistic effects [10,16,19]. However, the primary limitation of the double emulsion is that the system is thermodynamically unstable. This limitation is improved by reducing the droplet size from micrometer to nanometer scale. Furthermore, double nanoemulsion systems that require a smaller quantity of surfactants have better kinetic stability of particle aggregation and apparent optical clarity due to the size of the droplets [15,16]. The w/o/w nanoemulsions are commonly manufactured using two-step emulsification. The first process involves the homogenization of the water and oil phases with a hydrophobic surfactant. Consequently, the w/o/w is prepared by dispersing the w/o emulsion in an aqueous phase using a hydrophilic surfactant [14,15,16,19]. Presently, studies on natural health products and nutraceutical inventions prioritizing health promotion and reducing the risk factors of diseases are being carried out [20]. 

The objective of the present research was to show the feasibility of using a two-step high-energy emulsification technique to obtain a stable w/o/w nanoemulsion loaded with hydrophobic curcumin and hydrophilic betacyanin derived from temulawak and red dragon fruit extracts, respectively. The w/o/w nanoemulsion formulations were characterized based on their physicochemical properties, namely, w/o droplet size, stability, Fourier Transform Infrared (FTIR) spectra, zeta potential, antioxidant attributes, curcumin content, betacyanin content, and nanoemulsion morphology.

## 2. Results and Discussion

### 2.1. MDD and PDI

Table 1 summarizes the mean diameter of the w/o droplets dispersed in the outer water phase of the w/o/w nanoemulsions, which ranged from 180 to 247 nm. The effect of a decreasing concentration of w/o emulsion and an increasing concentration of Tween 80 on the decrease in MDD is statistically significant (*p* < 0.05). The higher concentration of hydrophilic emulsifier that led to a reduction in MDD was also reported by previous studies [18,21,22]. An increase in the emulsifier’s concentration in the external aqueous phase led to a reduction in the interfacial tension, which occurred due to the adsorption of some emulsifier into the interface. However, when the interface becomes saturated, the decreased interfacial tension reaches a relatively constant level [23].

Figure 1 shows the z-averaged w/o droplet size distribution of the w/o/w nanoemulsions (#8 and #13) as the least and the most stable formulation, respectively, showing a unimodal size distribution. The droplet size distribution of all of the nanoemulsions are shown in Figures S1–S14.

The nanoemulsions with the same concentration of w/o emulsion and a higher concentration of Tween 80 consistently produced a w/o emulsion with a smaller MDD (#8 < #9, #13 < #12). While, at the same concentration of emulsifier and a lower concentration of w/o emulsion, MDD had a tendency to decrease (#13 < #8, #12 < #9). The smallest droplet size of 189 nm was obtained using 15% w/w w/o emulsion and 1.5% *w*/*w* Tween 80. The composition, type of emulsifier used, and processing conditions had a significant effect on particle size in the double emulsion. The use of PGPR and Span 20 as hydrophobic emulsifiers aided in the formation of smaller droplet sizes in the w/o emulsion, which prevented flocculation and coalescence [24]. The effect of hydrophobic emulsifiers was not analyzed in this study.

The droplet distribution in the continuous phase of the w/o/w nanoemulsion is indicated by the PDI. The polydispersity of the w/o/w nanoemulsions is shown in Table 1, with values ranging from 0.15 to 0.22. A monodisperse population of nanoemulsion is usually represented by a PDI < 0.2 [16,19]. Nanoemulsion #3 (22.5 wt% of w/o emulsion and 0.89 wt% of Tween 80) and 7 (11.8 wt% of w/o emulsion and 1.25 wt% of Tween 80) showed PDI values of 0.22 and 0.22, respectively. The statistical analysis showed that the concentration of Tween 80 had a significant effect on the differences in the PDI values. The increase in Tween 80 caused a decrease in the PDI values (*p* < 0.05). This phenomenon is consistent with the results obtained from several studies [23,25].

Tween 80 (HLB of 15) is frequently used as an incorporated hydrophilic emulsifier while Span 20 (HLB of 8.6) and PGPR (HLB of 1.5) are utilized as hydrophobic emulsifiers in w/o/w double emulsions. This combination yields an effective HLB value [26], producing a w/o/w emulsion with excellent monodispersity. Tween 80 tends to produce micelles at higher concentrations, as well as enhances the solubility of the hydrophobic surfactants (PGPR and Span 20) [27]. It has been reported that the nature of the hydrophobic surfactants is the main factor used to obtain monodispersed w/o/w nanoemulsions [24,26,28].

### 2.2. Stability Study

A stable emulsion shows no perceptible transformation in droplet size distribution, aggregation state, and spatial arrangement over a certain period [24]. Several mechanisms that lead to the instability of w/o/w emulsions have been determined, such as, the coalescence of multiple emulsion droplets or internal aqueous droplets that causes phase separation, rupture of the oil layer on the surface of internal drops, shrinkage or swelling of internal droplets, flocculation of the internal aqueous phase, and multiple emulsion droplets [13,27]. A suitable stability test for a w/o/w nanoemulsion with applications in functional food should involve freezing and thawing steps, as many food emulsions are frozen to improve their shelf life [28,29]. The six cycles of freeze–thaw and the −4 °C freezing temperature used in the present study were selected to represent conditions during storage and demonstrate the use of the w/o/w nanoemulsion as a dietary supplement. 

Table 1 shows that all of the nanoemulsions subjected to the freeze–thaw stability test became unstable with varying degrees of oil phase separation that reached up to 20% *v*/*v*. In an o/w emulsion, the presence of interfacial stress may lead to the rupture of the oil phase during the water freezing phase and to the coalescence of the oil droplets during the thawing phase, thus forming a separate oil layer [28]. This explanation is consistent with the w/o/w nanoemulsion instability behavior observed in this study, where coalescence was enhanced at higher concentration of the o/w emulsion due to the proximity of the oil droplets. The most stable nanoemulsions (#7, #12, #13) with the least oil phase separation were those prepared using the least amount of w/o emulsion, while the least stable nanoemulsions (#4, #8) had the largest amount of w/o emulsion. In spite of this observed trend, the effect of w/o emulsion on the percentage of oil phase separation was not statistically significant (*p* > 0.05). A comparison of the three most stable nanoemulsions (#7, #12, #13) revealed that surfactant concentration only became an important factor in lowering the oil phase separation at a sufficiently low w/o emulsion concentration. The most stable nanoemulsion (#13) with an oil phase separation of only 5% *v*/*v* was obtained with a w/o emulsion concentration of 15% *w*/*w* and Tween 80 concentration of 1.5% *w*/*w*.

Notably, the oil phase separation of 5% *v*/*v*—20% *v*/*v* obtained in this study is significantly lower than the oil phase separation of 65% *w*/*w* reported for an o/w emulsion of Tween 80 (1% *w*/*w*)-stabilized corn oil (20% *w*/*w*) subjected to one cycle of freeze–thaw stability test at −20 °C for 22 h and at 25 °C for 2 h, respectively [29]. The large stability differences might be attributed to the presence of PGPR and Span 20 in the oil phase, along with Tween 80 in the outer water phase of the w/o/w nanoemulsions used in this study, which maintained the stability of the interface between the oil phase and the outer water phase during the freezing phase [30]. The increased stability of w/o emulsions with increasing PGPR and/or calcium concentrations has been reported previously [30], presumably due to smaller water droplets and a higher adsorption density of the emulsifier at the oil-outer water phase.

### 2.3. FTIR Spectroscopy

**Figure 2:**
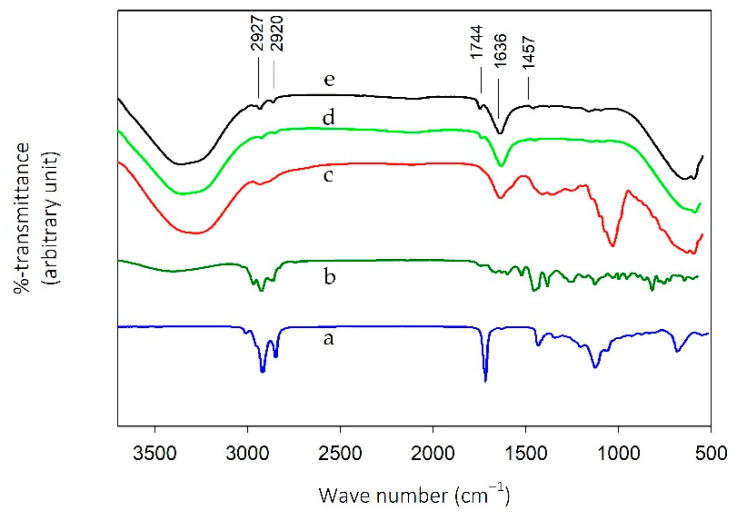
FTIR spectra of corn oil (**a**), temulawak extract (**b**), red dragon fruit extract (**c**), emulsion #13 (**d**), and emulsion #8 (**e**).

The FTIR spectra of corn oil, temulawak extract, red dragon fruit extract, and two of the w/o/w nanoemulsions are shown in Figure 2. Figure 2a is the FTIR spectrum of corn oil with a strong peak at 1744 cm^−1^ due to the C=O stretching absorption of the free fatty acids found in corn oil, oleic and linoleic acids [31,32]. Figure 2b represents the FTIR spectrum of the temulawak extract showing peaks that belong to curcumin, the -CH_3_ and -CH_2_- asymmetric stretching at 2961 cm^−1^ and 2921 cm^−1^ [33,34], and the -CH_3_ bending at 1450 cm^−1^ [35]. These peaks were observed at 2927 cm^−1^, 2920 cm^−1^, and 1457 cm^−1^ in Figure 2d (nanoemulsion #13) and 1e (nanoemulsion #8), an indication that temulawak extract was present in the w/o/w nanoemulsions. Figure 2c shows the FTIR spectrum of the red dragon fruit extract with a strong C=O stretching peak of betacyanin at 1636 cm^−1^, subsequently observed in the FTIR spectrum of the nanoemulsions [36]. The intensities of the peaks in Figure 2d were higher than those in Figure 2c due to the higher w/o emulsion concentration of 30% and 15% *w*/*w*, respectively.

### 2.4. Zeta Potential

The particle dispersed in an emulsion has a surface charge due to its attraction to a layer of oppositely charged particle; an electric double layer is thus generated [24]. An absolute value of zeta potential greater than 25 mV generally indicates a stable emulsion [21,37,38,39]. The zeta potentials of all nanoemulsions tested in this study were in the range –32.0 mV to –38.2 mV (Table 1), which means that the w/o emulsion droplets were negatively charged. It was concluded that these values are the stable potentials of all w/o/w nanoemulsions, as reported previously. As a non-ionic surfactant (Tween 80) was used to stabilize the w/o/w nanoemulsion, the negative zeta potential values could be attributed to the presence of the carboxylate functional group of linoleic and oleic acids in the oil phase, with pKa values of 9.2 and 9.8, respectively [40]. The presence of the fatty acids was verified by the carbonyl absorption peak in the FTIR spectra of the nanoemulsions. There were no statistically significant changes observed in the absolute value of zeta potential of all formulations (*p* ≥ 0.05). Nevertheless, the trend of zeta potential becoming less negative with increasing Tween 80 indicated that there was an electrostatic interaction between the surfactant and the fatty acids at the oil-external water interface.

### 2.5. Antioxidant Activity

The temulawak extract and the red dragon fruit extract contained 32.3% *w*/*w* curcumin and 0.15% *w*/*w* betacyanin, respectively. The IC_50_ value of the temulawak extract obtained through the DPPH assay was 91 ppm, which is slightly higher than 82 ppm [41] and 87 ppm [42] reported previously. Meanwhile, the IC_50_ value of the red dragon fruit extract was 925 ppm, which is higher than the reported value of 830 ppm [43]. These differences possibly occurred due to the various sources of temulawak and red dragon fruit extracts. The temulawak extract showed a much higher antioxidant activity compared with the red dragon fruit extract, as its IC_50_ value is about one-tenth lower.

The IC_50_ values of the nanoemulsions were in the range of 908–1073 ppm (mean value of 1014 ppm, standard deviation of 50 ppm), which did not increase with higher w/o emulsion content, or equivalently, with higher amount of curcumin. This observation indicated that the concentration of curcumin in the oil phase was not the only factor responsible for the overall antioxidant activity of the w/o/w nanoemulsions. A similar observation was ascribed to the interference of the other components of the nanoemulsion to the absorbance measurement, as DPPH scavenging is measured through visible spectroscopy [44]. The amount of added emulsifier could be important [45], however, Tween-80 has been shown to be inactive against the stable free radical DPPH [46]. The concentration of curcumin and betacyanin in nanoemulsion #13 was reduced to 1000 ppm and 3 ppm, respectively. In spite of that, the IC_50_ values of the nanoemulsions and the red dragon fruit extract are similar. It means that the higher concentration of curcumin in the nanoemulsions and the lower IC_50_ value of temulawak extract ensured sufficient antioxidant activities of the w/o/w nanoemulsions.

### 2.6. Morphology of W/O/W Nanoemulsions

**Figure 3:**
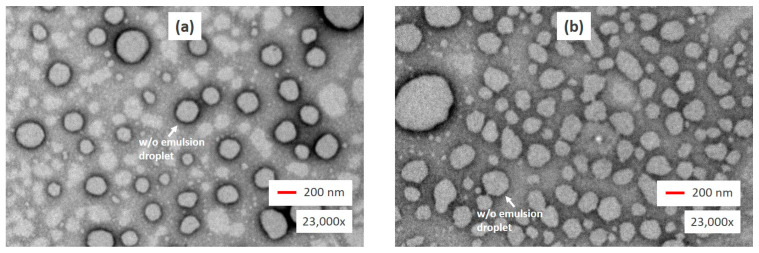
TEM images of the w/o/w nanoemulsions magnified 23,000×: sample 1 (**a**) and sample 9 (**b**).

Figure 3 shows the TEM images of the w/o/w nanoemulsions loaded with temulawak extract and red dragon fruit extract taken at a magnification of 23,000×. The TEM images of the two nanoemulsions are representative of the 14 nanoemulsion formulations listed in Table 1. The dynamic light scattering method yielded similar MDD and PDI for the two nanoemulsions: #1 (222 nm MDD, 0.19 PDI) and #9 (247 nm MDD, 0.20 PDI). The shape of the w/o droplet in Figure 3a appeared to be less round and regular than that in Figure 3b. These phenomena of surface shrinking and size reduction were made possible by the changes in surface tension, which was affected by the types and concentration of hydrophilic and hydrophobic surfactants. This finding is consistent with the results of previous studies [16,24,30]. The w/o oil droplets were uniformly dispersed in the external water phase. It was difficult to move the reverse micelle structures inside the oil droplets due to their small sizes [47]. 

## 3. Materials and Methods

### 3.1. Materials

Corn oil was used as an oil phase to prepare the w/o/w nanoemulsions (Mazola, ACH Food Companies, Inc, Oakbrook Terrace, IL, USA). The ethanolic temulawak and red dragon fruit extracts served as the hydrophobic and the hydrophilic compounds, respectively, which were encapsulated in the primary emulsion. The lipophilic emulsifiers consisted of Span 20 (Sigma-Aldrich, Singapore) and polyglycerol polyricinoleate (PGPR) (ZTCC, Henan Zhengtong Food Technology, Co. Ltd., Xingyang, China). Tween 80 (Merck KgaA, Darmstadt, Germany) served as a hydrophilic emulsifier. Curcumin (>95%) and betaine (>90%) standard, DPPH, and CaCl_2_ were purchased from Sigma Chemical Co, Singapore. All other chemicals were of analytical grade. The tools used included an Ultra Turrax, high-pressure homogenizer, particle size analyzer, transmission electron microscopy (TEM), UV spectroscopy, HPLC, FTIR, analytical scales, and laboratory glassware.

### 3.2. Preparation of Temulawak and Red Dragon Fruit Extracts

The dried powder obtained from the rhizomes of *C. xanthorriza* Roxb (1000 g) was macerated using 6000 mL of 96% ethanol overnight. The mixture was then heated at 50 °C and continuously stirred using a blade mixer at 150 rpm for 6 h. The acquired solution was filtered using several layers of cotton fabric. The final filtration was carried out using a fine Whatman filter paper. The filtrates were concentrated in a rotary evaporator at 40 °C to reduce the water content and residual solvent, after which they were stored in a refrigerator for further usage. The flesh of the red dragon fruit (1200 g) was crushed in a blender and then macerated using 6000 mL of 96% ethanol and was left overnight. The mixture was then heated at 50 °C and was continuously stirred with a blade mixer at 100 rpm for 5 h, after which it was allowed to cool. Subsequently, filtration was carried out using both cotton fabric and Whatman filter paper. The filtrates were then concentrated in a rotary evaporator at 40 °C and stored in a refrigerator for further usage.

### 3.3. Fabrication of w/o/w Nanoemulsions

The w/o/w nanoemulsions were fabricated using a two-step emulsification process to produce a w/o primary emulsion and a w/o/w nanoemulsion. The high-energy emulsification approaches that were referred to as the modified method reported in previous studies were applied in the two processes [15,16,25]. The factors that needed to be reviewed in the w/o/w emulsification were the concentrations of w/o (15% to 30% *w*/*w*) and Tween 80 (1% to 1.5% *w*/*w*) of the total w/o/w nanoemulsions. The central composite design, which was based on 14 runs, was used to determine the appropriate levels of independent variables, namely, the concentrations of primary emulsions and Tween 80. The dependent variables consisted of the mean droplet diameter (MDD), polydispersity index (PDI), zeta potential, and undamaged emulsion volume evaluated as a volume percentage of the stable emulsion.

#### 3.3.1. Primary w/o Emulsion

The primary w/o emulsion was prepared by mixing the inner aqueous phase (30% *v*/*v*) and corn oil (70% *v*/*v*). The oil phase contained the hydrophobic emulsifiers PGPR (2% *w*/*w*) and Span 20 (1% *w*/*w*). The temulawak extract (3% *w*/*w*) was dissolved in the oil phase at 40 °C using a magnetic stirrer at 350 rpm for 10 min. The inner aqueous phase contained red dragon fruit extract (5% *w*/*w*) and CaCl_2_ (0.3% *w*/*w*) dissolved using a magnetic stirrer at 350 rpm for 5 min. The w/o emulsions were prepared by mixing the oil and inner aqueous phases using a high shear mixer (Ultra-Turrax T 24 Basic, IKA, Staufen, Germany) at 13,000 rpm for 5 min.

#### 3.3.2. Final w/o/w Nanoemulsion

The second step emulsification process was carried out using a high-pressure homogenizer (GEA Niro Soavi, type Panda plus 2000, Parma, Italy) operated at 250–300 bar. The w/o/w nanoemulsions were prepared by gradually adding a primary emulsion (15–30% *w*/*w*) into the external aqueous phase containing Tween 80 (1–1.5% *w*/*w*). The mixture was fed into the homogenizer, and the emulsification in the homogenizer was repeated for three cycles.

### 3.4. Stability Study

The stability of the double w/o/w nanoemulsion was determined by freezing and thawing all of the formulations for six cycles. The mixtures were placed in a 15 mL tube and kept in a freezer at –4 °C for 22 h and were subsequently thawed in an incubator chamber at 40 °C for 2 h. After six cycles, the volume of the separated oil phase was recorded.

### 3.5. Droplet Size Measurement

The MDD and PDI of the multiple droplet sizes were measured through dynamic light scattering (Zetasizer Nano-ZS, Malvern Instruments Ltd., Malvern, UK). First, the double w/o/w nanoemulsion was diluted in aquadest (1:200). All measurements were carried out at a temperature of 25 °C and a fixed scatter angle of 173 and reported as mean ± standard deviation (SD) of a triplicate measurement (*n* = 3). The PDI was a dimensionless measure of the size distribution width, determined by the cumulant analysis ranging from 0 to 1.

### 3.6. Zeta Potential Measurement

The zeta potential of the w/o droplets was the measurement result of electrophoretic mobility using a Zetasizer Nano-ZS (Malvern Instruments Ltd., Malvern, UK). The samples were diluted at a ratio of 1:200 in aquadest. The measured zeta potentials at 25 °C were reported as mean ± SD (*n* = 3).

### 3.7. Antioxidant Activity

The antioxidant attributes of the temulawak extract, red dragon fruit extract, and w/o/w nanoemulsions were evaluated through DPPH radical-scavenging assay, referred to as the modified method [8,48]. The DPPH solution (0.2 mM) was prepared using ethanol (80% *v*/*v*). Up to 50 µL of the solution was added to 150 µL of the w/o/w nanoemulsion, after which the resulting solution was mixed and incubated in a dark room for 30 min at 25 °C. The degradation of DPPH was determined spectrophotometrically at 517 nm. The free radical-scavenging activity was calculated using the following equation:

(1) $$DPPH\;scavenging\;effect\%=\left(1-\frac{A_{S}}{A_{C}}\right)\times 100$$

where *A_C_* and *A_S_* are the absorbance of the control and the sample, respectively. The 50% inhibitory concentration (IC_50_) of DPPH radical scavenging activity of each sample was determined using its calibration curve.

### 3.8. FTIR Spectra

The FTIR spectra of the temulawak extract, red dragon fruit extract, and all w/o/w nanoemulsions were obtained using a Nicolet iS50 FTIR spectrometer (Thermo Scientific, Waltham, MA, USA). This tool is equipped with a KBr beam splitter and a DTGS KBr detector. The spectra were recorded between 400 and 4000 cm^−1^ at a resolution of 2 cm^−1^.

### 3.9. TEM Images

The TEM images of the w/o/w nanoemulsions were obtained using an FEI Tecnai G2 Spirit Twin Transmission electron microscope (Tecnai Ltd., Geleen, The Netherlands) operated at an accelerating voltage of 120 kV. The w/o/w nanoemulsions were diluted eight times with aquadest, and 20 µL of the diluted solution was applied to a 400-mesh copper grid (support film formvar or carbon 400 mesh). The grid was kept under ambient conditions for 1 min. The excess sample was absorbed using Whatman 41 filter paper. Approximately 2% of staining uranyl acetate, which served as a negative staining agent, was applied to the grid and left to dry before the TEM images were taken.

## 4. Conclusions

This study shows the feasibility of using w/o/w nanoemulsions as a delivery system of temulawak extract and red dragon fruit extract as lipophilic and hydrophilic nutraceuticals, respectively, through a two-step emulsification method. The most stable nanoemulsion was obtained using w/o emulsion concentration of 15% *w*/*w* and Tween 80 concentration of 1.5% *w*/*w* with 5% *v*/*v* oil phase separation, 189 nm MDD, 0.16 PDI, and –32 mV zeta potential. The freeze–thaw stability might be attributed to the combination of a low w/o emulsion content and a high Tween 80 concentration in the outer water phase of the w/o/w nanoemulsions used in this study. The FTIR spectra show that the temulawak extract and red dragon fruit extracts are present in the oil and aqueous phases, respectively, while the TEM images show the morphology of the double emulsion. The IC_50_ values of the nanoemulsion and the red dragon fruit extract are similar, which means that the higher concentration of curcumin in the nanoemulsions and the lower IC_50_ value of temulawak extract ensured sufficient antioxidant activities of the w/o/w nanoemulsions.

## Figures and Tables

**Figure 1:**
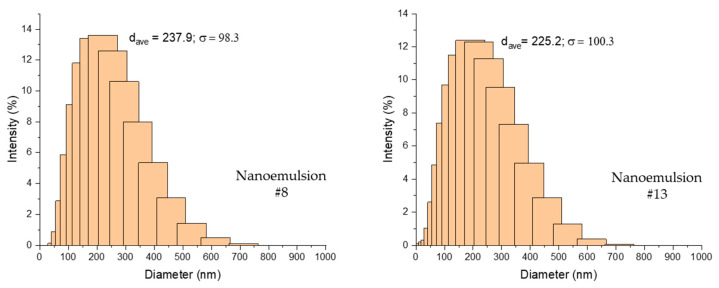
Z-averaged droplet size distribution of the least (# 8) and the most stable (#13) w/o/w nanoemulsions.

**Table 1:** Characterization of w/o/w nanoemulsions.

Emulsion # ^1^	w/o Emulsion (% *w*/*w*)	Tween 80 (% *w*/*w*)	MDD ^2^ (nm)	PDI ^2^	Zeta Potential (mV)	Oil Phase Separation ^3^ (% *v*/*v*)
1	22.5	1.25	222 ± 3	0.19	−37.0 ± 2.5	15
2	22.5	1.25	207 ± 1	0.17	−34.2 ± 1.4	15
3	22.5	0.89	243 ± 2	0.22	−38.2 ± 4.0	15
4	33.1	1.25	216 ± 1	0.15	−33.2 ± 1.2	20
5	22.5	1.60	180 ± 1	0.15	−34.0 ± 2.5	15
6	22.5	1.25	195 ± 1	0.17	−32.7 ± 0.9	15
7	11.9	1.25	191 ± 2	0.22	−35.0 ± 0.9	10
8	30.0	1.50	209 ± 2	0.17	−34.2 ± 0.9	20
9	30.0	1.00	247 ± 1	0.20	−35.1 ± 3.3	15
10	22.5	1.25	197 ± 1	0.15	−32.6 ± 0.7	15
11	22.5	1.25	204 ± 1	0.16	−32.3 ± 0.6	15
12	15.0	1.00	203 ± 2	0.19	−34.5 ± 2.1	10
13	15.0	1.50	189 ± 2	0.16	−32.0 ± 1.1	5
14	22.5	1.25	219 ± 2	0.19	−34.2 ± 1.5	15

^1^ Emulsions #1, 2, 6, 10, 11, and 14 are the center points of the central composite design. ^2^ MDD and PDI are properties of the w/o emulsions. Each value of MDD and zeta potential represents the mean ± SD (*n* = 3). ^3^ Indicates stability of the w/o/w nanoemulsion after six cycles of freezing (−4 °C, 22 h) and thawing (40 °C, 2 h).

## Supplementary Materials

The following are available online, Figures S1–S14: Droplet size distribution of nanoemulsion #1–#14.

## Sample Availability

Samples are not available from the authors.
