# Supplementary material for: Water-in-Oil-in-Water Nanoemulsions Containing Temulawak (Curcuma xanthorriza Roxb) and Red Dragon Fruit (Hylocereus polyrhizus) Extracts
Source: Molecules. 2021 Jan 2;26(1):196. doi: 10.3390/molecules26010196 (PMC7795868; doi:10.3390/molecules26010196)
Supplement: Supplementary file 1 [file molecules-26-00196-s001.pdf]

Supporting Information

Water-in-Oil-in-Water Nanoemulsions Containing Temulawak (*Curcuma xanthorriza* Roxb) and Red Dragon Fruit (*Hylocereus polyrhizus*) Extracts

Niken Harimurti, Mohammad Nasikin, and Kamarza Mulia

			Size (d.nm):	% Intensity:	St Dev (d.n...
Z-Average (d.nm):	222.2	Peak 1:	269.8	98.8	126.0
PdI:	0.191	Peak 2:	56.65	0.7	8.616
Intercept:	0.947	Peak 3:	4633	0.5	814.1
Result quality :	Good				

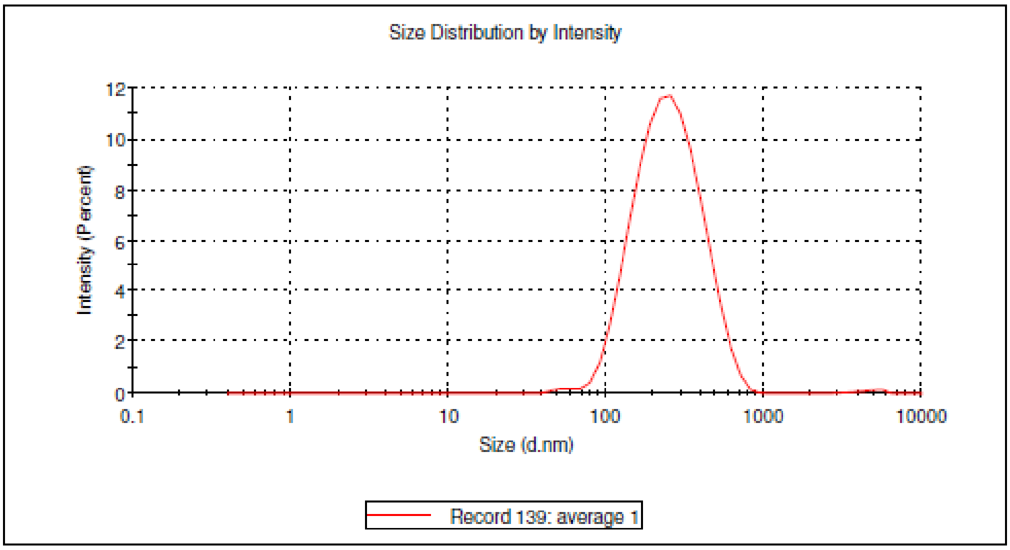

Figure S1: Droplet size distribution report: Nanoemulsion #1

			Size (d.nm):	% Intensity:	St Dev (d.n...
Z-Average (d.nm):	206.6	Peak 1:	236.7	98.9	104.4
PdI:	0.168	Peak 2:	4763	1.1	759.2
Intercept:	0.971	Peak 3:	0.000	0.0	0.000
Result quality :	Good				

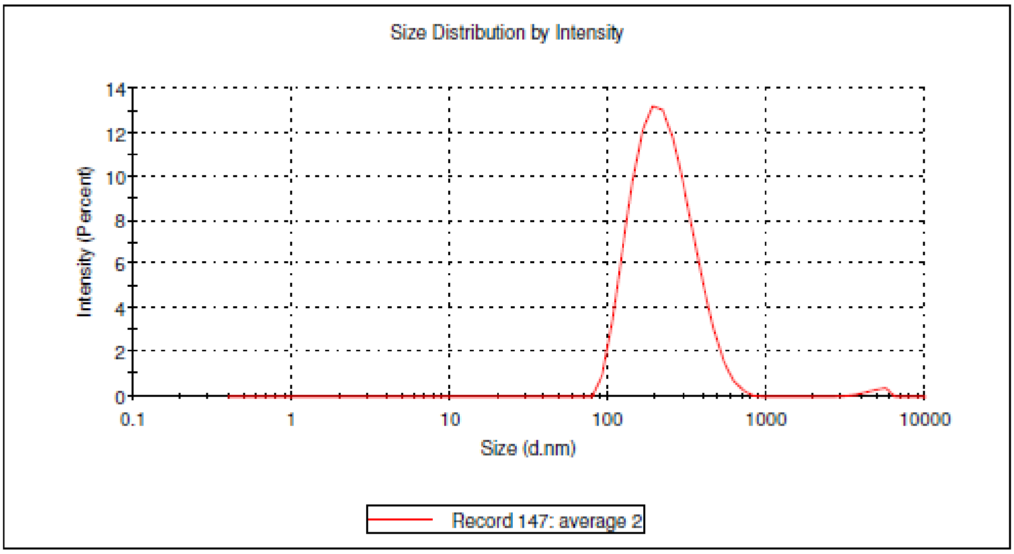

Figure S2: Droplet size distribution report: Nanoemulsion #2

			Size (d.nm):	% Intensity:	St Dev (d.n...
Z-Average (d.nm):	243.4	Peak 1:	303.8	98.9	159.3
PdI:	0.215	Peak 2:	4783	1.1	738.1
Intercept:	0.958	Peak 3:	0.000	0.0	0.000
Result quality :	Good				

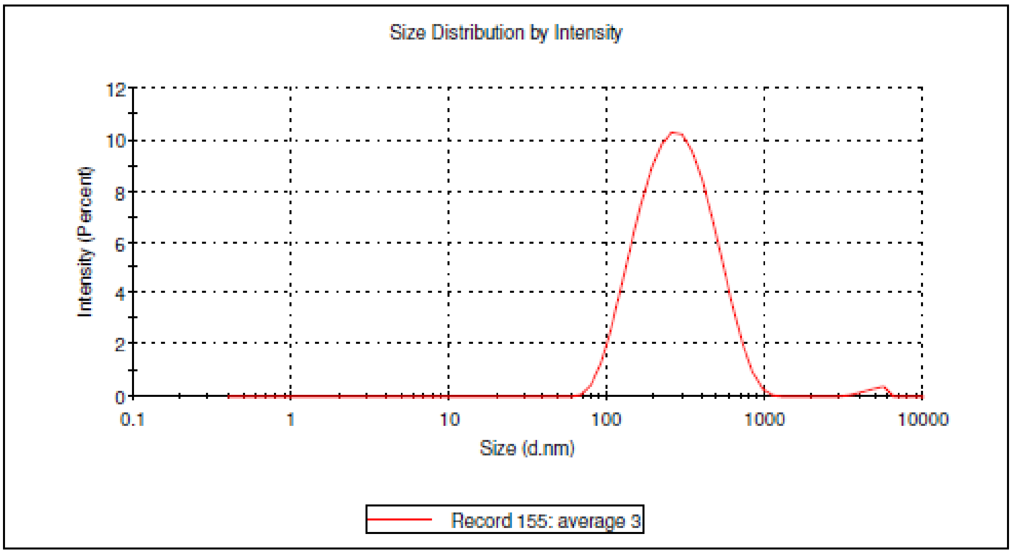

Figure S3: Droplet size distribution report: Nanoemulsion #3

			Size (d.nm):	% Intensity:	St Dev (d.n...
Z-Average (d.nm):	215.6	Peak 1:	244.9	99.4	101.3
PdI:	0.149	Peak 2:	5036	0.6	588.1
Intercept:	0.975	Peak 3:	0.000	0.0	0.000
Result quality :	Good				

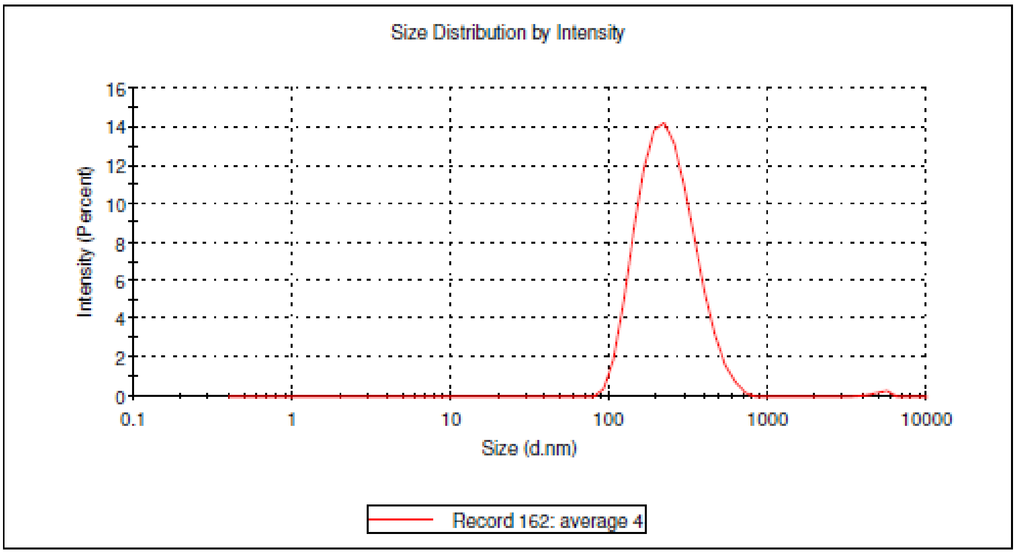

Figure S4: Droplet size distribution report: Nanoemulsion #4

			Size (d.nm):	% Intensity:	St Dev (d.n...
Z-Average (d.nm):	180.4	Peak 1:	203.4	100.0	78.90
PdI:	0.149	Peak 2:	0.000	0.0	0.000
Intercept:	0.974	Peak 3:	0.000	0.0	0.000
Result quality :	Good				

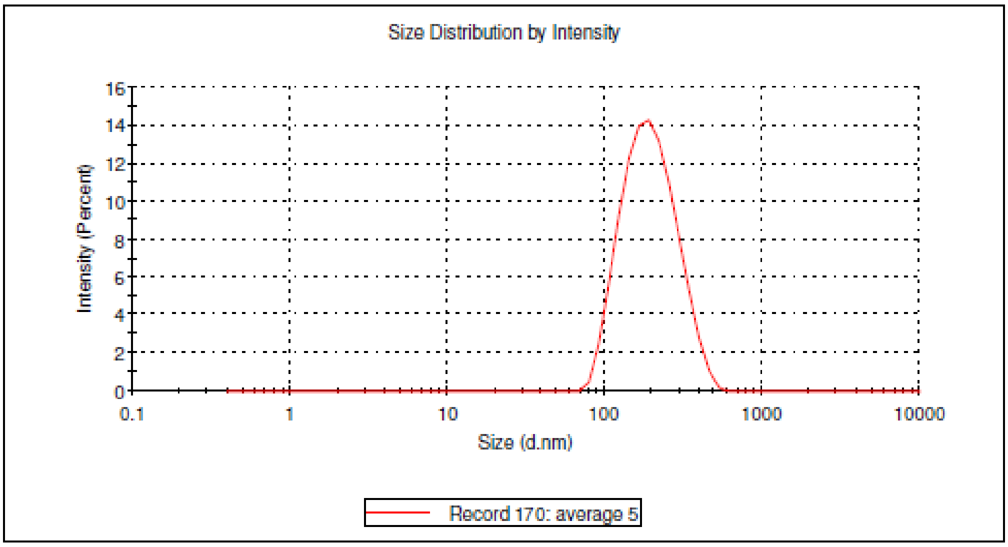

Figure S5: Droplet size distribution report: Nanoemulsion 5

			Size (d.nm):	% Intensity:	St Dev (d.n...
Z-Average (d.nm):	195.2	Peak 1:	218.0	98.9	86.38
PdI:	0.165	Peak 2:	4987	1.1	618.3
Intercept:	0.975	Peak 3:	0.000	0.0	0.000
Result quality :	Good				

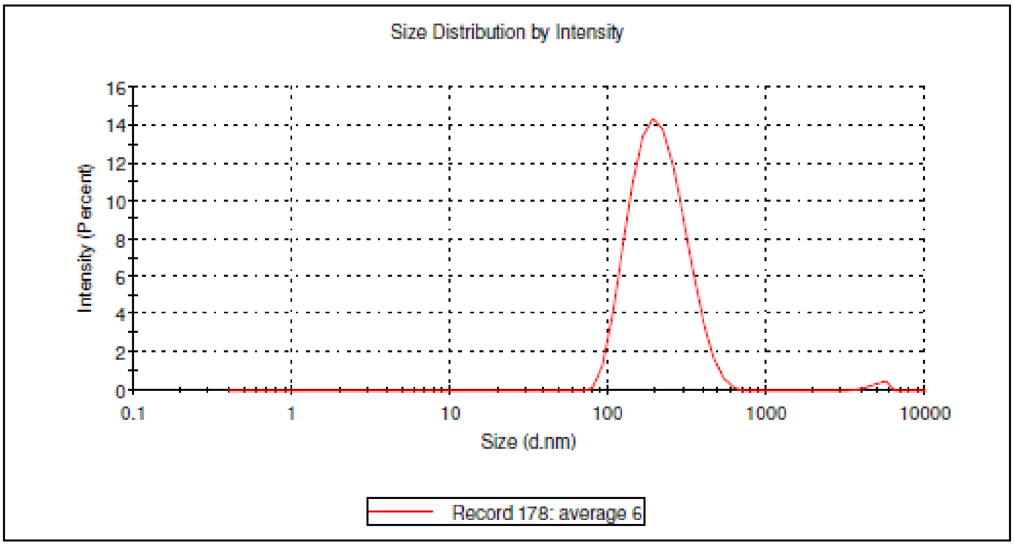

Figure S6: Droplet size distribution report: Nanoemulsion #6

			Size (d.nm):	% Intensity:	St Dev (d.n...
Z-Average (d.nm):	191.1	Peak 1:	216.7	95.4	100.3
PdI:	0.219	Peak 2:	1723	3.3	741.8
Intercept:	0.949	Peak 3:	4875	1.3	691.7
Result quality :	Good				

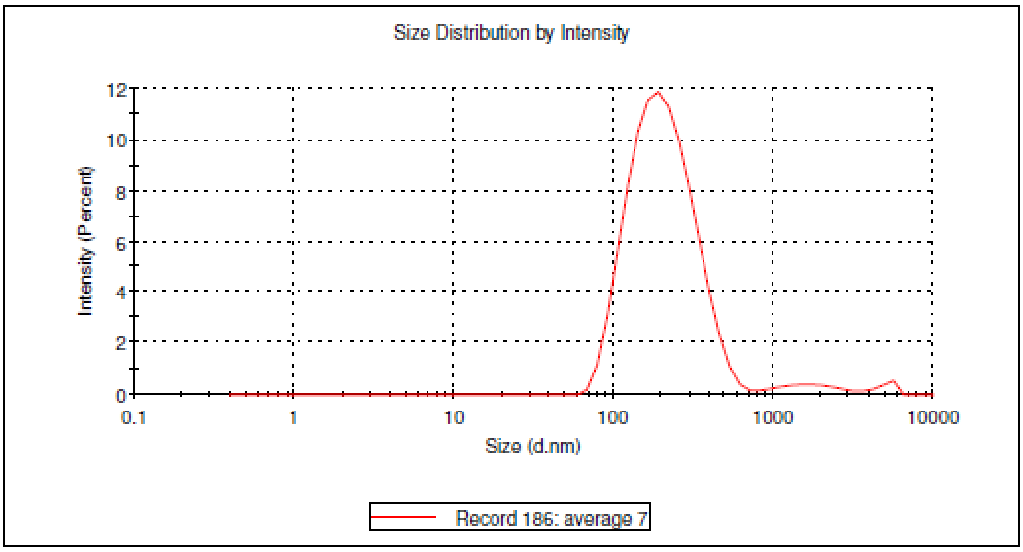

Figure S7: Droplet size distribution report: Nanoemulsion #7

			Size (d.nm):	% Intensity:	St Dev (d.n...
Z-Average (d.nm):	209.5	Peak 1:	237.9	99.3	98.27
PdI:	0.166	Peak 2:	4895	0.7	672.0
Intercept:	0.976	Peak 3:	0.000	0.0	0.000
Result quality :	Good				

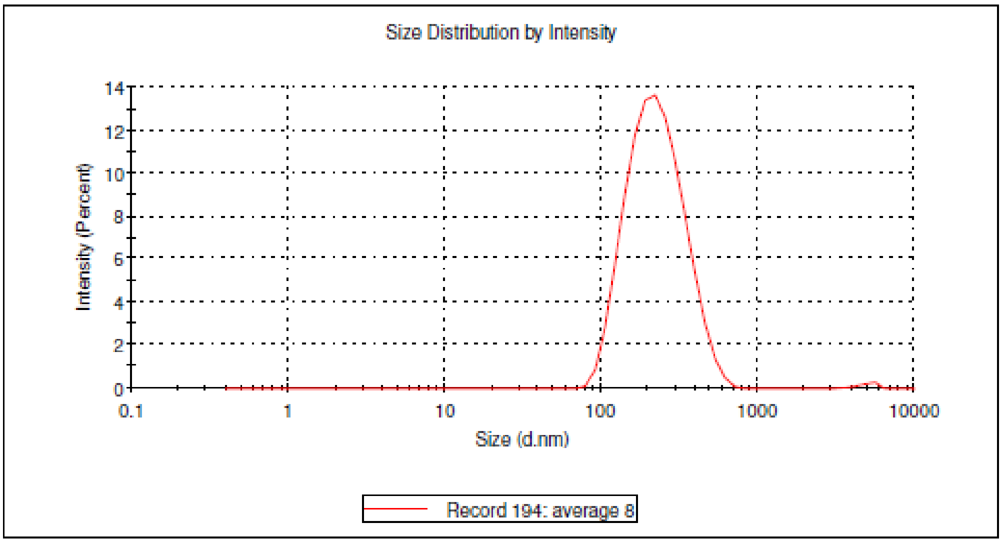

Figure S8: Droplet size distribution report: Nanoemulsion #8

			Size (d.nm):	% Intensity:	St Dev (d.n...
Z-Average (d.nm):	246.8	Peak 1:	297.4	97.7	155.2
PdI:	0.195	Peak 2:	3468	2.3	1324
Intercept:	0.980	Peak 3:	0.000	0.0	0.000
Result quality :	Good				

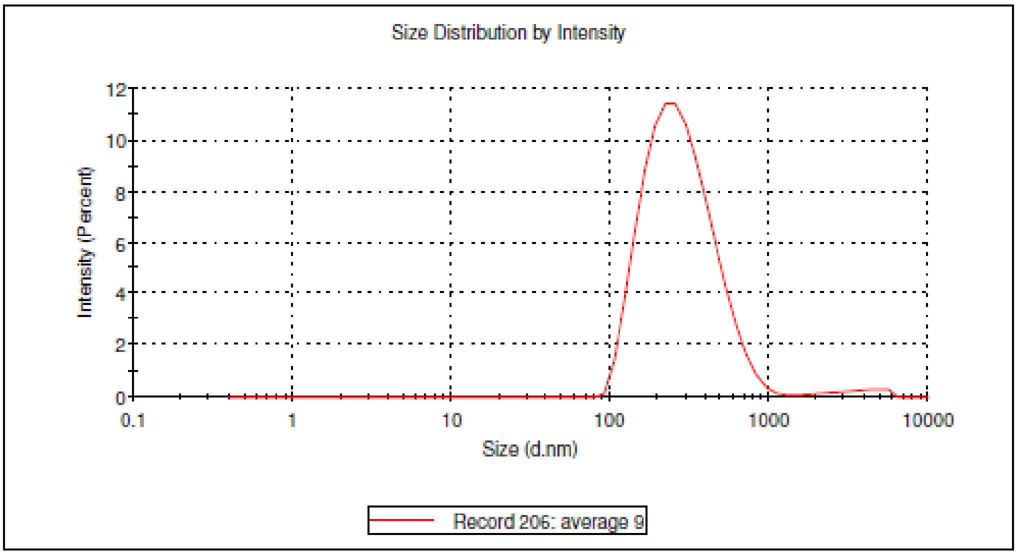

Figure S9: Droplet size distribution report: Nanoemulsion #9

			Size (d.nm):	% Intensity:	St Dev (d.n...
Z-Average (d.nm):	197.5	Peak 1:	230.5	99.6	90.98
PdI:	0.145	Peak 2:	54.36	0.4	8.549
Intercept:	0.971	Peak 3:	0.000	0.0	0.000
Result quality :	Good				

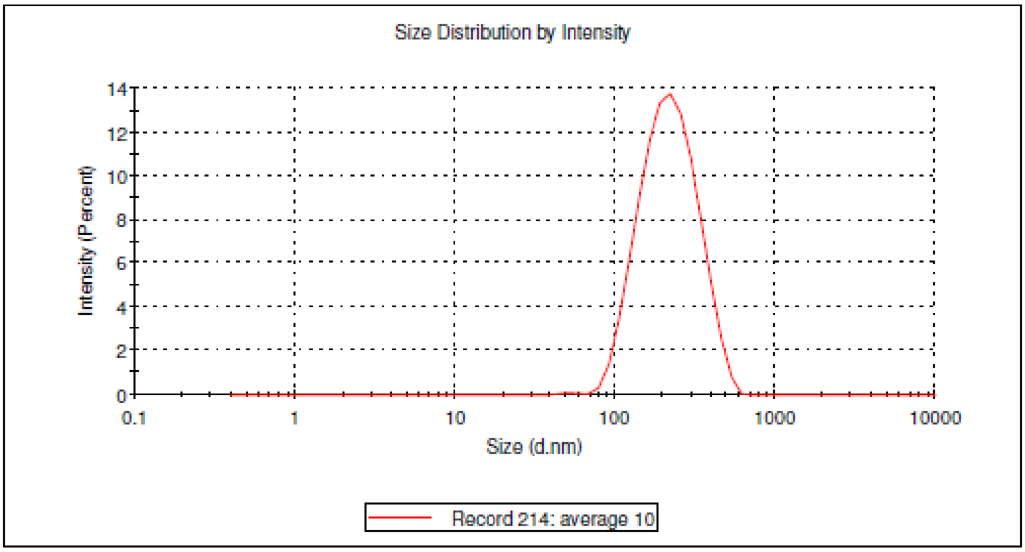

Figure S10: Droplet size distribution report: Nanoemulsion #10

			Size (d.nm):	% Intensity:	St Dev (d.n...
Z-Average (d.nm):	204.5	Peak 1:	225.4	98.8	84.11
PdI:	0.157	Peak 2:	4950	1.2	661.3
Intercept:	0.974	Peak 3:	0.000	0.0	0.000
Result quality :	Good				

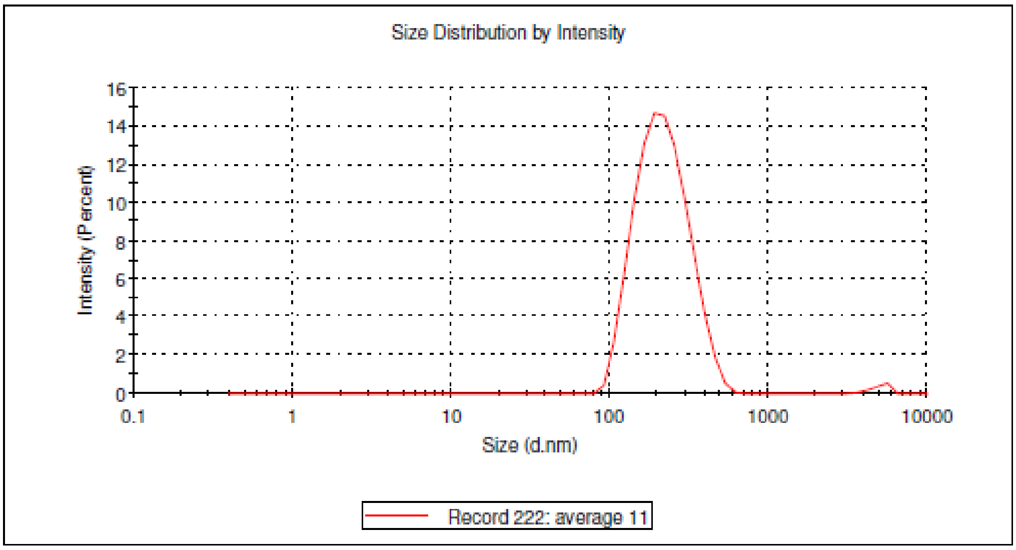

Figure S11: Droplet size distribution report: Nanoemulsion #11

			Size (d.nm):	% Intensity:	St Dev (d.n...
Z-Average (d.nm):	203.0	Peak 1:	250.3	100.0	125.9
PdI:	0.189	Peak 2:	0.000	0.0	0.000
Intercept:	0.945	Peak 3:	0.000	0.0	0.000
Result quality :	Good				

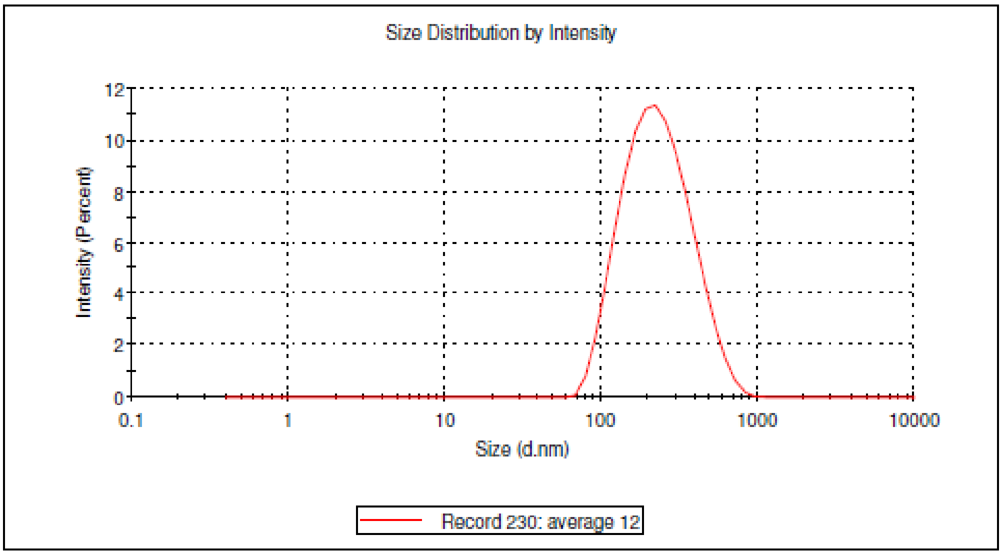

Figure S12: Droplet size distribution report: Nanoemulsion #12

			Size (d.nm):	% Intensity:	St Dev (d.n...
Z-Average (d.nm):	189.2	Peak 1:	225.2	100.0	100.3
PdI:	0.161	Peak 2:	0.000	0.0	0.000
Intercept:	0.946	Peak 3:	0.000	0.0	0.000

Result quality : Good

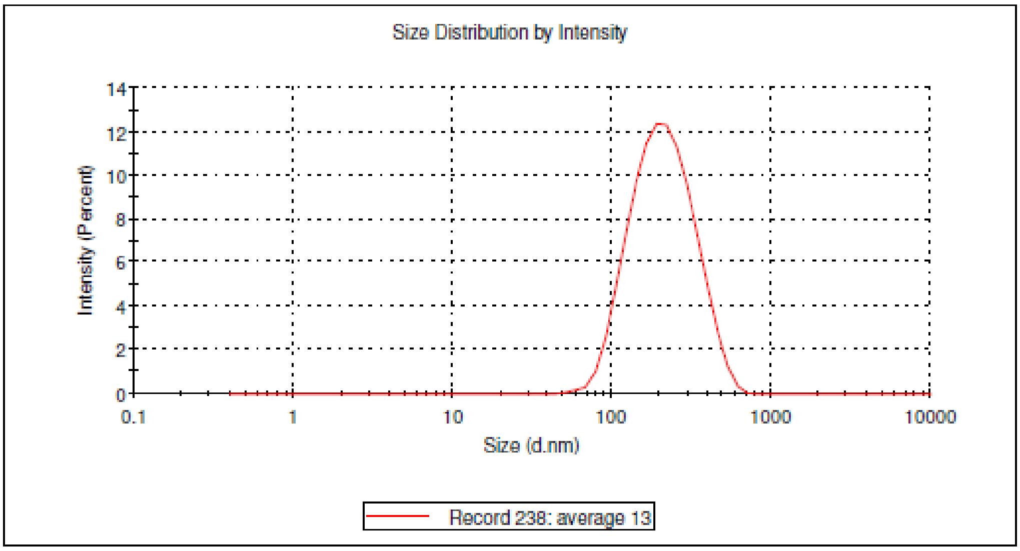

Figure S13: Droplet size distribution report: Nanoemulsion #13

			Size (d.nm):	% Intensity:	St Dev (d.n...
Z-Average (d.nm):	219.0	Peak 1:	270.7	99.5	139.5
PdI:	0.192	Peak 2:	4841	0.5	704.8
Intercept:	0.978	Peak 3:	0.000	0.0	0.000

Result quality : Good

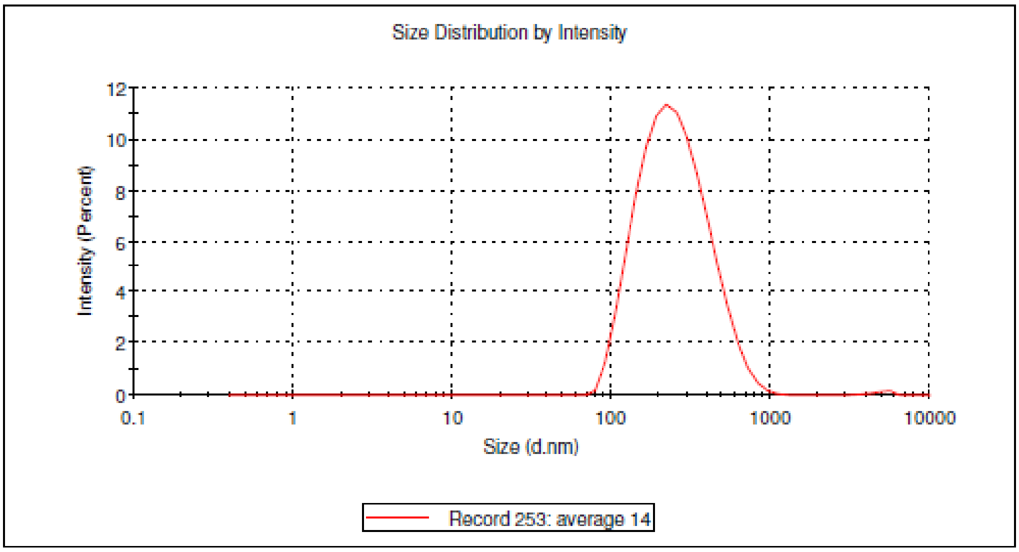

Figure S14: Droplet size distribution report: Nanoemulsion #14
